# Dialysis vintage modifies the effect of adsorption-based therapies on protein-bound toxin clearance

**DOI:** 10.3389/fcell.2026.1856227

**Published:** 2026-05-29

**Authors:** Peng Zhang, Liyuan Ma, Bo Zhao, Rui Hao, Xuan Wang, Li Yuan, Zenan Niu, Shudong Zhang

**Affiliations:** Department of Nephrology, The Second Clinical Medicine School of Ningxia Medical University (The First People’s Hospital of Yinchuan), Yinchuan, Ningxia, China

**Keywords:** dialysis vintage, hemodialysis, hemoperfusion, protein-bound uremic toxins, restricted cubic spline

## Abstract

**Introduction:**

The efficiency of hemodialysis varies substantially across different solute classes, but the determinants of this variability remain poorly characterized, limiting personalized prescription.

**Methods:**

In this prospective randomized study, 60 maintenance hemodialysis patients were assigned to three modalities: hemodiafiltration (HDF), hemodialysis with HA130 hemoperfusion, and hemodialysis with KHA130 hemoperfusion. Clearance of p-cresyl sulfate (PCS), β2-microglobulin (β2-MG), advanced glycation end products (AGEs), and indoxyl sulfate (IS) was assessed. Multivariable linear regression, interaction analysis, subgroup analysis stratified by dialysis vintage tertiles, nonlinear quadratic regression, and machine learning (LASSO and Elastic Net) were applied.

**Results:**

HD + KHA130 significantly improved PCS clearance compared with HDF (β = 45.26, 95% CI: 6.26-84.27, P = 0.023), an effect robust to multivariable adjustment. Significant interactions between treatment modality and dialysis vintage were observed for PCS (P = 0.008), β2-MG (P = 0.002), and IS (P = 0.044). Exploratory subgroup analyses suggested that adsorption may benefit β2-MG and IS more in short dialysis vintage, whereas HD + KHA130 appeared more advantageous for PCS in long vintage patients. The relationship between dialysis vintage and PCS clearance followed a U-shaped pattern, with a nadir at approximately 60 months (bootstrap median; 95% CI: 9.6-218.1; P = 0.038). Machine learning identified dialysis vintage, age, treatment method, and CRP as the most stable predictors of PCS clearance.

**Conclusion:**

HD + KHA130 significantly enhances PCS clearance, and may be most beneficial in patients with longer dialysis vintage. These findings support personalized treatment selection based on both solute type and dialysis vintage.

## Introduction

Maintenance hemodialysis (MHD) remains the cornerstone of life-sustaining therapy for end-stage renal disease (ESRD), with its primary objective being the effective removal of accumulated uremic toxins (Shi and Fan, 2025). Traditionally, hemodialysis mainly relies on the diffusion principle and has a relatively high efficiency in removing small molecule water-soluble toxins (such as urea and creatinine), but its ability to remove middle molecules (with molecular weights ranging from 0.5 to 60 kDa) and protein-bound uremic toxins (PBUTs) is extremely limited (Cozzolino et al., 2025; Maduell et al., 2025). In recent years, with the deepening understanding of uremic syndrome, the continuous accumulation of PBUTs and specific middle molecules are closely related to the occurrence and development of long-term complications in MHD patients, including accelerated atherosclerotic cardiovascular diseases, progressive malnutrition-inflammation-atherosclerosis syndrome, dialysis-related amyloidosis, cognitive dysfunction and depression (Ito and Yoshida, 2014). Consequently, achieving optimal clearance across the entire spectrum of uremic toxins, rather than merely targeting urea reduction, has emerged as a critical goal in contemporary nephrology practice.

To address these limitations, technological advancements have focused on augmenting dialysis efficacy through modalities such as hemodiafiltration (HDF) and the integration of adsorptive therapies, including resin- or cartridge-based adsorption columns (Zhang et al., 2021; Wang et al., 2025). HDF enhances the clearance of intermediate molecular substances through the principle of convection (Canaud et al., 2025). Hemoperfusion, especially the HA130 with neutral microporous resin adsorbent, due to its specific adsorption ability for PBUTs and inflammatory factors, is widely used in clinical practice in China (Li et al., 2017; Yu et al., 2025). While these approaches hold theoretical promise for enhancing the removal of broader solute spectra, clinical evidence remains inconsistent (Duval-Sabatier et al., 2023; Biedunkiewicz et al., 2024), and the factors determining their effectiveness are poorly understood. In particular, the role of baseline patient characteristics in modulating clearance outcomes has not been systematically investigated across different solute classes.

The emergence of machine learning offers a means to overcome these limitations by identifying complex, non-linear predictors of solute-specific clearance (Gómez-Pulido et al., 2021; Ravindhran et al., 2023; Li et al., 2025). Therefore, this study aimed to: (1) evaluate associations between baseline patient characteristics and clearance of PCS, β2-MG, IS and AGEs; (2) develop multivariate predictive models for each solute class; (3) apply machine learning to identify robust determinants of clearance efficacy; and (4) assess the modifying effects of dialysis modalities, especially adsorptive therapies. By clarifying these determinants, we seek to inform personalized dialysis strategies aimed at improving solute control and outcomes in ESRD.

## Materials and methods

### Study design and participants

This prospective, randomized, single-center study was conducted following the principles of the Declaration of Helsinki and was approved by the institutional ethics committee. All participants provided written informed consent. Eligible patients were randomly assigned (1:1:1) to one of three treatment groups using a computer-generated randomization sequence. Allocation concealment was achieved using sequentially numbered, opaque, sealed envelopes. The study enrolled stable MHD patients (dialysis vintage >3 months) in the hospital from May 2023 to May 2025. Inclusion criteria: age ≥18 years old; thrice-weekly dialysis regimen. Exclusion criteria: known hypersensitivity or intolerance to the dialyzer, hemoperfusion cartridge, or anticoagulant; platelet count <60 × 10^9^/L; blood flow <200 mL/min; serum albumin <30 g/L; coagulopathy, severe bleeding tendency, or active bleeding; hypotension or severe cardiopulmonary dysfunction; concurrent participation in another interventional trial; or presence of acute infection, serious cardiac, pulmonary, hepatic, or neurological diseases, or malignancy. Laboratory personnel responsible for measuring toxin concentrations (PCS, IS, β2-MG, AGEs) and outcome assessors were blinded to treatment group assignment. The randomization code was kept by an independent statistician not involved in patient recruitment or outcome assessment. Due to the visible differences in dialysis machines and hemoperfusion cartridges, patients and treating physicians could not be blinded to the treatment modality.

### Treatment protocols

Participants were categorized into three treatment groups based on their prescribed dialysis modality.

1. HDF group: Received online post-dilution hemodiafiltration using a high-flux dialyzer (Fresenius, Germany), with a blood flow of 230–290 mL/min and a total substitution volume of 20–24 L;
2. HD + HA group: Underwent high-flux hemodialysis (Hips15, Fresenius, Germany) combined with a HA130 hemoperfusion cartridge (Jafron Biomedical, Zhuhai, China) placed in series before the dialyzer. Hemoperfusion was performed at 150 mL/min for 2 h, after which the cartridge was removed and hemodialysis continued at 200–250 mL/min for an additional 2 h;
3. HD + KHA group: Followed the same protocol as the HD + HA group, but using a KHA130 hemoperfusion cartridge.

To account for the different blood flow profiles across treatment groups, a time-weighted average blood flow was calculated for each patient. For the HDF group, which maintained a constant blood flow of 230–290 mL/min throughout the 4-h session, the time-weighted blood flow equaled the recorded blood flow. For the HD + HA130 and HD + KHA130 groups, where blood flow was set at 150 mL/min during the first 2 h (hemoperfusion phase) and then increased to 200–250 mL/min for the remaining 2 h (hemodialysis phase), the time-weighted blood flow was calculated as (150 × 2 + recorded blood flow × 2)/4. All groups used bicarbonate dialysate (Ca 1.25–1.5 mmol/L, dialysate flow 500 mL/min) and low-molecular-weight heparin (60–80 U/kg) for anticoagulation. Anticoagulation status was monitored by clinical observation of the extracorporeal circuit.

### Data collection and laboratory measurements

Demographic and clinical characteristics (dialysis vintage, primary disease, comorbidities), and treatment parameters (blood flow, treatment time, anticoagulant dose) were recorded. Venous blood samples were collected via vascular access before and 4 h after the dialysis session. Complete blood counts (white blood cells [WBC], hemoglobin [Hb], platelets [PLT]) were analyzed using an automated hematology analyzer. Renal function and electrolytes (blood urea nitrogen [BUN], serum creatinine [Scr], uric acid [UA], calcium [Ca], phosphorus [P], iron [Fe], kalium [K]), liver function and lipid profiles (total bilirubin [TBIL], triglyceride [TG], total cholesterol [TC], low-density lipoproteins [LDL], high-density lipoproteins [HDL]) were measured with an automated biochemistry analyzer. Albumin (ALB) and pre-albumin (PAB) were determined by immunoturbidimetry. Serum ferritin [SF], transferrin saturation [TSAT] were assessed via chemiluminescence immunoassay and calculation (serum iron/total iron-binding capacity ×100%), respectively. C-reactive protein (CRP) was measured by high-sensitivity immunoturbidimetry. Advanced glycation end products (AGEs), indoxyl sulfate (IS), and p-cresyl sulfate (PCS) were quantified using ultra-performance liquid chromatography-tandem mass spectrometry with isotope-labeled internal standards. β2 microglobulin (β2-MG) and intact parathyroid hormone (iPTH) were analyzed by fluorescence quantitative assays. Tumor necrosis factor-α (TNF-α) and interleukin-6 (IL-6) were measured via enzyme-linked immunosorbent assay.

### Outcome definition

The primary outcomes were the absolute clearances of four solutes (PCS, IS, AGEs and β2-MG), calculated as the pre-dialysis concentration minus the post-dialysis concentration.

### Statistical analysis

Multiple imputation with predictive mean matching (m = 5 imputations) was applied to address missing data, and results were pooled using Rubin’s rules. Baseline characteristics across the three treatment groups were compared using the Kruskal–Wallis test for continuous variables and Fisher’s exact test for categorical variables, with false discovery rate (FDR) correction for multiple comparisons. Univariate linear regression models were fitted for each clearance outcome, and variables with *P* < 0.10 were considered candidates for further analysis.

Treatment effects were assessed using Kruskal–Wallis test, with unadjusted effect sizes quantified by Cohen’s d and Hedges’ g for pairwise comparisons. To account for potential baseline imbalances, adjusted effect sizes were calculated using ANCOVA with age, gender, and ALB as covariates. Adjusted effect sizes were reported as partial η^2^ with 95% confidence intervals obtained from 500 bootstrap resamples. Effect size interpretation followed standard thresholds: for Cohen’s d, 0.2 (small), 0.5 (medium), 0.8 (large); for partial η^2^, 0.01 (small), 0.06 (medium), 0.14 (large).

Model robustness was validated via 500-iteration bootstrap resampling. Potential effect modification by dialysis vintage was examined by including an interaction term in the multivariable linear regression models adjusted for age, gender, and ALB. Subgroup analyses stratified by dialysis vintage tertiles were performed using ANCOVA adjusting for age, gender, and ALB to compare clearance across treatment groups.

To explore nonlinear associations, restricted cubic spline (RCS) models with three knots (at the fifth, 50th, and 95th percentiles) and quadratic polynomial models were fitted, with nonlinearity tested by joint assessment of spline coefficients. Model fit was compared using Akaike Information Criterion (AIC) and Bayesian Information Criterion (BIC). The nadir of the quadratic curve and its 95% confidence interval were estimated using bootstrap resampling (1,000 iterations).

For exploratory feature selection, LASSO regression (α = 1) and Elastic Net (α = 0.5) were applied, both tuned via leave-one-out cross-validation. Given the modest sample size, stability of selected features was assessed by bootstrap resampling (n = 200) for Elastic Net, with a predefined stability threshold of >80% selection frequency to identify robust predictors.

Several sensitivity analyses were conducted to assess the robustness of the findings. Extreme values (≥3 standard deviations) were excluded to evaluate the influence of outliers. ANCOVA models adjusting for age, gender and ALB were fitted to confirm the independence of treatment effects. Sensitivity analyses additionally adjusted for time-weighted blood flow and TNF-α to address potential confounding from baseline imbalances; these yielded consistent effect size estimates. Bootstrap resampling (500 iterations) was performed to obtain 95% confidence intervals for the primary adjusted effect size. Post-hoc power analysis was conducted to determine the statistical power to detect the observed effect size with the current sample size. To assess whether the observed U-shaped relationship between dialysis vintage and PCS clearance could be explained by mathematical coupling or measurement error, we performed a sensitivity analysis by adding random noise (5% of the measured value) to pre-dialysis PCS concentrations and recalculated absolute clearance (500 iterations). The quadratic model was refitted for each iteration, and the distribution of quadratic coefficients, nadir estimates, and significance rates was examined. All analyses were performed in R (version 4.3.2) with a two-tailed significance level of *P* < 0.05.

## Results

### Baseline characteristics and solute clearance patterns

**FIGURE 1:**
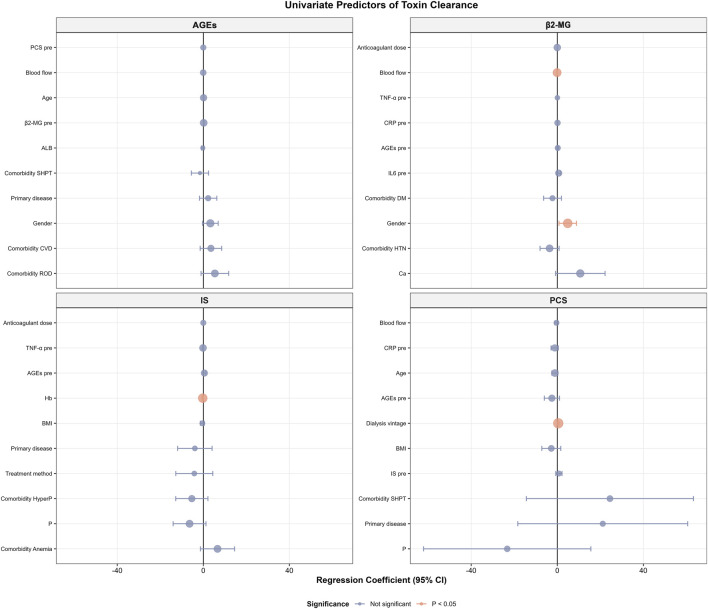
Univariate predictors of solute clearance. Forest plots display regression coefficients (β) and 95% confidence intervals (CI) for predictors of clearance of four uremic solutes: advanced glycation end products (AGEs), β_2_-microglobulin (β2-MG), indoxyl sulfate (IS), and p-cresyl sulfate (PCS). Blue circles: non-significant associations (*P* ≥ 0.05). Orange circles: Statistically significant associations (*P* < 0.05). The vertical dashed line at β = 0 indicates no association. Positive coefficients denote higher predictor values associated with increased solute clearance, while negative coefficients denote higher predictor values associated with decreased solute clearance.

A total of 60 patients were enrolled, with 20 patients in each treatment group. Baseline characteristics were well-balanced across groups after false discovery rate (FDR) correction. Unadjusted differences in hypertension (*P* = 0.025), TNF-α (*P* = 0.031), and blood flow (*P* = 0.006) did not remain significant after FDR adjustment (*P* > 0.05). Detailed baseline characteristics are presented in Table 1. Univariate analyses identified distinct correlates for each clearance outcome (Figure 1; Supplementary Table S1). PCS clearance was associated with dialysis vintage (β = 0.455, *P* = 0.007). β2-MG clearance showed associations with gender (β = 4.855, *P* = 0.019) and blood flow (β = −0.087, *P* = 0.039). AGEs clearance demonstrated marginal associations with gender (β = 3.268, *P* = 0.078). IS clearance was significantly associated with Hb (β = −0.264, *P* = 0.020).

**TABLE 1:** Baseline characteristics of study participants.

Variable	HDF (n = 20)	HD + HA130 (n = 20)	HD + KHA130 (n = 20)	*P*	FDR
Age (years)	61.50 (48.50, 66.25)	53.50 (45.50, 59.00)	52.50 (46.5, 60.00)	0.225	0.711
Gender, Male	11 (55.00%)	13 (65.00%)	13 (65.00%)	0.841	0.986
BMI (kg/m^2^)	23.95 (21.58, 25.80)	21.60 (20.88, 24.83)	24.20 (22.43, 26.28)	0.406	0.832
Dialysis vintage (months)	62.00 (34.25, 91.50)	29.50 (24.00, 91.50)	47.50 (25.50, 123.50)	0.598	0.975
Primary disease, secondary	14 (70.00%)	15 (75.00%)	15 (75.00%)	1.000	1.000
Comorbidity					
DM	10 (50.00%)	8 (40.00%)	9 (45.00%)	0.946	1.000
HTN	10 (50.00%)	18 (90.00%)	14 (70.00%)	0.025	0.425
HyperP	8 (40.00%)	5 (25.00%)	7 (35.00%)	0.704	0.986
Anemia	13 (65.00%)	15 (75.00%)	16 (80.00%)	0.667	0.986
SHPT	2 (10.00%)	7 (35.00%)	8 (40.00%)	0.089	0.503
ROD	0 (0.00%)	4 (20.00%)	1 (5.00%)	0.117	0.503
CVD	4 (20.00%)	2 (10.00%)	3 (15.00%)	0.900	1.000
Blood flow (mL)	250 (220, 250)	220 (220, 222)	220 (220, 220)	0.006	0.232
Anticoagulant dose (U)	3250 (2500, 3850)	3300 (3000, 4075)	3500 (3500, 4000)	0.195	0.667
WBC (10^9^/L)	5.99 (4.52, 8.12)	5.85 (4.33, 6.43)	6.30 (4.25, 7.70)	0.819	0.986
Hb (g/L)	116.00 (108.00, 121.00)	111.50 (108.50, 125.00)	105.00 (93.75, 116.25)	0.123	0.503
PLT (10^9^/L)	210.00 (134.00, 240.75)	155.00 (124.25, 190.50)	155.00 (135.00, 206.00)	0.379	0.832
ALB (g/L)	39.82 (37.38, 42.55)	40.25 (38.71, 42.44)	39.42 (37.36, 40.49)	0.726	0.986
PAB (mg/L)	446.89 (371.71, 497.71)	464.38 (381.07, 527.59)	417.39 (365.38, 519.79)	0.796	0.986
BUN (mmol/L)	20.11 (18.05, 22.99)	20.85 (17.75, 25.48)	20.69 (18.62, 24.07)	0.715	0.986
Scr (μmol/L)	802.40 (620.95, 1,043.28)	961.35 (813.98, 1,060.27)	856.80 (694.20, 960.10)	0.351	0.832
UA (μmol/L)	357.10 (335.88, 400.97)	353.45 (322.98, 433.65)	358.90 (335.75, 416.85)	0.955	1.000
TBIL (μmol/L)	7.32 (5.95, 8.09)	8.09 (5.47, 9.81)	6.50 (5.15, 8.37)	0.396	0.832
TG (mmol/L)	2.21 (1.08, 2.64)	1.37 (1.05, 1.91)	1.32 (0.91, 2.40)	0.265	0.732
TC (mmol/L)	4.13 (3.67, 4.54)	3.37 (3.15, 3.95)	3.62 (3.07, 4.39)	0.101	0.503
LDL (mmol/L)	2.42 (2.20, 2.96)	2.02 (1.69, 2.42)	2.23 (1.57, 2.69)	0.118	0.503
HDL (mmol/L)	0.95 (0.90, 1.10)	0.99 (0.93, 1.06)	0.92 (0.84, 1.01)	0.470	0.876
Ca (mmol/L)	2.19 (2.14, 2.32)	2.27 (2.12, 2.35)	2.21 (2.10, 2.31)	0.609	0.975
P (mmol/L)	1.38 (1.23, 1.69)	1.60 (1.34, 1.87)	1.60 (1.35, 1.70)	0.520	0.926
Fe (μmol/L)	11.10 (8.22, 13.68)	14.05 (10.83, 17.92)	11.10 (9.40, 14.55)	0.268	0.732
K (mmol/L)	4.70 (4.06, 5.31)	4.99 (4.46, 5.30)	4.62 (4.40, 5.46)	0.460	0.876
iPTH (pg/mL)	15.35 (10.18, 34.92)	21.45 (11.73, 30.10)	19.00 (13.28, 32.08)	0.618	0.975
SF (ng/mL)	234.00 (56.07, 544.00)	207.00 (112.20, 391.50)	205.50 (71.90, 580.50)	0.801	0.986
TSAT (%)	20.37 (11.90, 29.05)	18.35 (13.07, 31.03)	20.88 (12.14, 29.17)	0.922	1.000
IL-6 pre (pg/mL)	9.07 (7.25, 10.61)	9.23 (8.25, 10.99)	9.09 (7.05, 10.50)	0.799	0.986
TNF-α pre (pg/mL)	71.24 (57.49, 86.18)	90.66 (76.14, 102.02)	67.14 (56.09, 77.44)	0.031	0.425
CRP pre (mg/L)	3.90 (1.48, 6.65)	2.30 (1.60, 10.25)	3.10 (1.70, 5.93)	0.982	1.000
β2-MG pre (mg/L)	40.82 (35.17, 45.05)	39.45 (36.06, 43.24)	43.97 (38.33, 46.21)	0.360	0.832
IS pre (μg/mL)	36.73 (33.19, 45.50)	38.67 (35.22, 40.74)	47.15 (34.60, 59.71)	0.177	0.660
AGEs pre (μg/mL)	27.12 (22.53, 28.58)	24.27 (18.53, 27.94)	23.12 (18.57, 26.07)	0.123	0.503
PCS pre (ng/mL)	168.27 (147.68, 230.75)	181.52 (137.42, 228.53)	249.41 (168.25, 288.44)	0.064	0.503

Data are presented as median (IQR) for continuous variables and n (%) for categorical variables. *P*-values were calculated using Kruskal–Wallis test for continuous variables and Fisher’s exact test for categorical variables. Adjusted *P*-values were corrected for false discovery rate (FDR). Abbreviations: HDF: hemodiafiltration; HD: hemodialysis; HA130: hemoadsorber HA130; KHA130: modified hemoadsorber KHA130; BMI: body mass index; DM: diabetes; HTN: hypertension; HyperP: hyperphosphatemia; SHPT: secondary hyperparathyroidism; ROD: renal osteodystrophy; CVD: cardiovascular disease; WBC: white blood cell; Hb: hemoglobin; PLT: platelets; ALB: albumin; PAB: prealbumin; BUN: blood urea nitrogen; Scr: serum creatinine; UA: uric acid; TBIL: total bilirubin; TG: triglyceride; TC: total cholesterol; LDL: low-density lipoproteins; HDL: high-density lipoproteins; Ca: calcium; P: phosphorus; Fe: iron; K: kalium; iPTH: intact parathyroid hormone; SF: serum ferritin; TSAT: transferrin saturation; IL-6: interleukin-6; TNF-α: tumor necrosis factor-α; CRP: C-reactive protein; β2-MG: β2 microglobulin; IS: indoxyl sulfate; AGEs: advanced glycation end-products; PCS: p-cresol sulfate.

### Treatment effects on solute clearance

**FIGURE 2:**
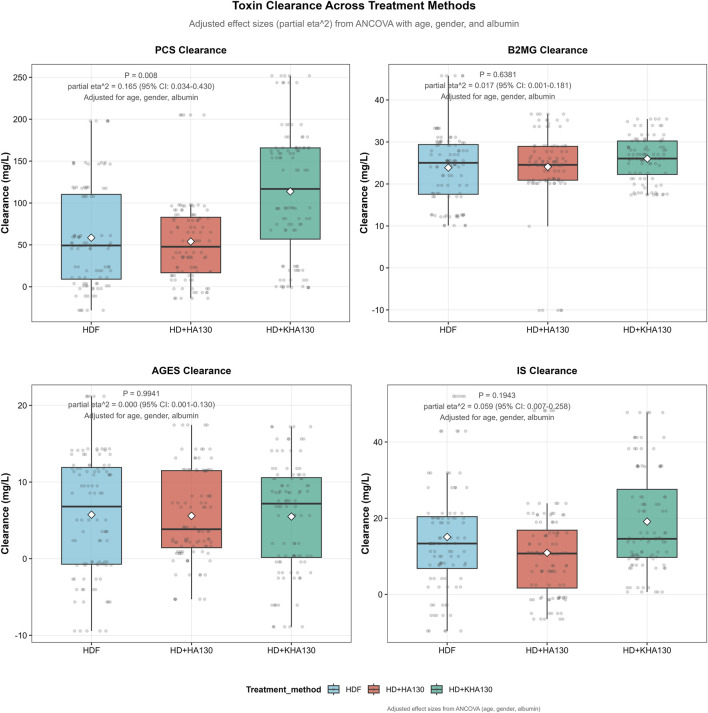
Solute clearance by treatment method. Box plots with overlaid individual data points show the distribution of clearance for four uremic solutes: p-cresyl sulfate (PCS), β_2_-microglobulin (β2-MG), advanced glycation end products (AGEs), and indoxyl sulfate (IS), across three treatment groups: hemodiafiltration (HDF, blue), hemodialysis + HA130 (HD + HA130, red), and hemodialysis + KHA130 (HD + KHA130, green). The box represents the interquartile range (IQR, 25th-75th percentiles), the horizontal line inside the box denotes the median, and the diamond marker indicates the mean. Whiskers extend to the minimum and maximum values within 1.5×IQR. Overall group differences are quantified by *P* values and partial eta-squared (η^2^) from ANCOVA models adjusted for age, gender, and ALB. Effect size interpretation: η^2^ ≥ 0.01 (small), ≥0.06 (medium), ≥0.14 (large). Pairwise comparisons are presented as adjusted effect sizes (partial η^2^ converted to Cohen’s d scale) for visual consistency. The conversion formula is: Cohen’s d = √(η^2^/(1-η^2^)) × 2. Significance is denoted based on Cohen’s d thresholds: *: d ≥ 0.2; **: d ≥ 0.5; ***: d ≥ 0.8.

The results of treatment effects revealed a significant difference only for PCS clearance (*P* = 0.008). Unadjusted pairwise comparisons demonstrated a large effect size between HD + HA130 and HD + KHA130 (Cohen’s d = −0.902, 95% CI: −1.55 to −0.25), and a medium effect between HDF and HD + KHA130 (Cohen’s d = −0.780, 95% CI: −1.42 to −0.14). No significant treatment effects were observed for β2-MG, AGEs, or IS clearance (Figure 2; Table 2). To account for potential confounding due to baseline differences in blood flow and TNF-α, we calculated adjusted effect sizes using ANCOVA with age, gender, and ALB as covariates. After adjustment, the effect of HD + KHA130 on PCS clearance remained substantial, with a partial η^2^ of 0.165 (95% CI: 0.032–0.407), corresponding to a large effect size (Table 2; Supplementary Figure S1).

**TABLE 2:** Treatment effect estimates for solute clearance.

Toxin	Comparison	Unadjusted Cohen’s d (95% CI)	Adjusted partial η^2^ (95% CI)
PCS	HDF vs. HD + HA130	−0.123 (−0.74, 0.50)	0.009 (0.000–0.112)
	HDF vs. HD + KHA130	−0.780 (−1.42, −0.14)	0.165 (0.032–0.407)
	HD + HA130 vs. HD + KHA130	−0.902 (−1.55, −0.25)	0.165 (0.032–0.407)
β2-MG	HDF vs. HD + HA130	−0.234 (−0.85, 0.39)	0.036 (0.001–0.158)
	HDF vs. HD + KHA130	−0.156 (−0.77, 0.46)	0.016 (0.000–0.106)
AGEs	HDF vs. HD + HA130	0.089 (−0.53, 0.70)	0.005 (0.000–0.085)
	HDF vs. HD + KHA130	0.234 (−0.38, 0.85)	0.034 (0.001–0.152)
IS	HDF vs. HD + HA130	−0.245 (−0.86, 0.38)	0.039 (0.001–0.163)
	HDF vs. HD + KHA130	−0.167 (−0.78, 0.45)	0.018 (0.000–0.112)

Adjusted for age, gender, and ALB., η^2^ interpretation: <0.01 = very small, 0.01-0.06 = small, 0.06-0.14 = medium, ≥0.14 = large. 95% confidence intervals were obtained from 500 bootstrap resamples. For pairwise comparisons, partial η^2^ is reported for the overall treatment effect (all three groups) rather than individual pairwise contrasts, as ANCOVA, provides a single effect size for the factor. The value shown is the overall partial η^2^ for PCS, clearance (0.165), which applies to all pairwise comparisons.

### Multivariable analysis

After adjusting for age, gender and univariately significant covariates (time-weighted blood flow and Ca for β2-MG; Hb for IS), a significant overall treatment effect observed only for PCS clearance (Supplementary Table S2, F = 5.843, *P* = 0.005). Specifically, HD + KHA130 remained superior to HDF (β = 45.26, 95% CI: 6.26-84.27, *P* = 0.023), while HD + HA130 did not differ from HDF (β = −9.58, 95% CI: −49.24-30.08, *P* = 0.633). When dialysis vintage was included as a predictor, it showed a significant positive linear association with PCS clearance (β = 0.40, 95% CI: 0.08-0.71, *P* = 0.013). For IS clearance, Hb was identified as a negative predictor (β = −0.27, 95% CI: −0.52 to −0.02, *P* = 0.035, Table 3). No significant treatment effects or independent predictors were identified for β2-MG or AGEs clearance. Variance inflation factors were below two for all models, indicating no substantial multicollinearity (Supplementary Table S3).

**TABLE 3:** Multivariable linear regression analysis for solute clearance.

Outcome	Predictor	β (95% CI)	*P*	FDR
PCS clearance				
​	Treatment method (ref: HDF)	​	​	​
​	HD + HA130	−9.58 (−48.87, 29.72)	0.633	0.633
​	HD + KHA130	45.26 (6.26, 84.27)	0.023	0.053
​	Age	−0.98 (−2.32, 0.35)	0.148	0.222
​	Gender (Female vs. Male)	−11.76 (−44.36, 20.84)	0.480	0.575
​	Dialysis vintage	0.40 (0.08, 0.71)	0.013	0.053
β2-MG clearance				
​	Treatment method (ref: HDF)	​	​	​
​	HD + HA130	0.71 (−4.22, 5.64)	0.779	0.839
​	HD + KHA130	2.90 (−2.00, 7.80)	0.246	0.839
​	Age	0.01 (−0.17, 0.18)	0.951	0.951
​	Gender (Female vs. Male)	3.82 (−0.40, 8.05)	0.076	0.373
​	Time-weighted blood flow	−0.08 (−0.17, 0.01)	0.093	0.373
​	Ca	8.54 (−3.43, 20.50)	0.162	0.432
AGEs clearance				
​	Treatment method (ref: HDF)	​	​	​
​	HD + HA130	0.77 (−3.74, 5.27)	0.739	0.927
​	HD + KHA130	0.54 (−3.93, 5.01)	0.813	0.927
​	Age	0.09 (−0.06, 0.25)	0.231	0.694
​	Gender (Female vs. Male)	2.93 (−0.83, 6.69)	0.126	0.694
IS clearance				
​	Treatment method (ref: HDF)	​	​	​
​	HD + HA130	−4.42 (−13.18, 4.34)	0.323	0.603
​	HD + KHA130	2.09 (−6.86, 11.03)	0.647	0.910
​	Age	−0.04 (−0.34, 0.26)	0.806	0.910
​	Gender (Female vs. Male)	0.43 (−7.08, 7.94)	0.910	0.910
​	Hb	−0.27 (−0.52, −0.02)	0.035	0.124

Models adjusted for age, gender, and univariately significant covariates (time-weighted blood flow and Ca for β2-MG; Hb for IS). HDF, served as the reference group for treatment method. β: unstandardized regression coefficient; CI: confidence interval; FDR: false discovery rate. Dialysis vintage was included as a predictor only for PCS, based on univariate significance (P = 0.007). Dialysis vintage was not included as a covariate for other outcomes.

### Interaction with dialysis vintage

After adjustment for age, gender, and ALB, significant interactions between treatment method and dialysis vintage were observed for PCS (*P* = 0.008), β2-MG (*P* = 0.002), and IS clearance (*P* = 0.044), but not for AGEs clearance (*P* = 0.579, Table 4). For PCS clearance, the HD + KHA130 × dialysis vintage coefficient was positive but did not reach statistical significance (β = 0.581, 95% CI: −0.262 to 1.424, *P* = 0.177). For β2-MG and IS clearance, both adsorption therapies demonstrated significant negative interactions with dialysis vintage (β2-MG: HD + HA130 β = −0.238, 95% CI: −0.351 to −0.125, *P* < 0.001; HD + KHA130 β = −0.130, 95% CI: −0.230 to −0.030, *P* = 0.010; IS: HD + HA130 β = −0.235, 95% CI: −0.451 to −0.019, *P* = 0.034; HD + KHA130 β = −0.283, 95% CI: −0.475 to −0.091, *P* = 0.004), indicating that the clearance advantage of adsorption-based therapies diminished with increasing dialysis vintage.

**TABLE 4:** Interaction analysis between treatment modality and dialysis vintage.

Outcome	Interaction term	β (95% CI)	*P*	Overall interaction *P*
PCS clearance	​	​	​	0.008
​	HD + HA130 × dialysis vintage	−0.357 (−1.310, 0.596)	0.463	​
​	HD + KHA130 × dialysis vintage	0.581 (−0.262, 1.424)	0.177	​
β2-MG clearance	​	​	​	0.002
​	HD + HA130 × dialysis vintage	−0.238 (−0.351, −0.125)	<0.001	​
​	HD + KHA130 × dialysis vintage	−0.130 (−0.230, −0.030)	0.010	​
AGEs clearance	​	​	​	0.579
​	HD + HA130 × dialysis vintage	−0.075 (−0.191, 0.041)	0.201	​
​	HD + KHA130 × dialysis vintage	−0.043 (−0.145, 0.059)	0.415	​
IS clearance	​	​	​	0.044
​	HD + HA130 × dialysis vintage	−0.235 (−0.451, −0.019)	0.034	​
​	HD + KHA130 × dialysis vintage	−0.283 (−0.475, −0.091)	0.004	​

Models adjusted for age, gender, and ALB. HDF, served as the reference group for treatment method. Overall interaction *P*-values were derived from comparing models with and without interaction terms. β: unstandardized regression coefficient; CI: confidence interval.

### Subgroup analysis by dialysis vintage

**FIGURE 3:**
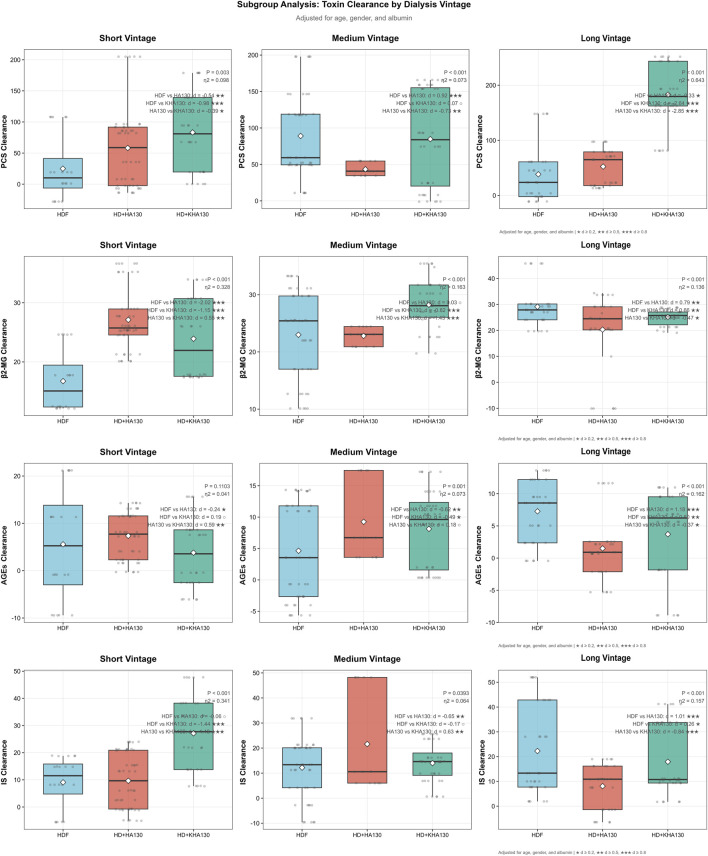
Subgroup analysis of solute clearance by dialysis vintage. Box plots with overlaid individual data points display the distribution of clearance for four uremic solutes (A: PCS, B: β2-MG, C: AGEs, D IS) across three treatment groups (HDF: blue; HD + HA130: red; HD + KHA130: green), stratified by dialysis vintage tertiles (Short, Medium, Long). All analyses are adjusted for age, gender, and serum ALB. The box represents the interquartile range (IQR, 25th–75th percentiles), the horizontal line inside the box denotes the median, and the diamond marker indicates the adjusted mean. Whiskers extend to the minimum and maximum values within 1.5×IQR. *P* values and partial eta-squared (η^2^) quantify the overall treatment effect within each vintage subgroup. Cohen’s d effect sizes are provided for each treatment contrast, with significance denoted as: *: d ≥ 0.2; **: d ≥ 0.5; ***: d ≥ 0.8.

Given the limited sample size, the following subgroup analyses are exploratory and hypothesis-generating. Stratified by dialysis vintage tertiles using ANCOVA (Figure 3), treatment effects appeared to vary across dialysis vintage. For PCS clearance, significant treatment effects were strongest in the long vintage group (*P* < 0.001), where HD + KHA130 outperformed HDF (*P* < 0.001). For β2-MG clearance, effects were observed in the short vintage group (*P* < 0.001), where both adsorption therapies outperformed HDF (*P* < 0.001). For AGEs clearance, significant effects emerged in the medium (*P* = 0.001) and long vintage groups (*P* < 0.001), with both adsorption therapies outperforming HDF (*P* < 0.01), and only HD + HA130 in the long group (*P* = 0.034). For IS clearance, effects were observed in the short vintage group (*P* < 0.001), where HD + KHA130 outperformed HDF (*P* < 0.001). These exploratory findings suggest that the efficacy of adsorption-based therapies may vary by dialysis vintage, with β2-MG and IS clearance potentially benefiting patients with shorter vintage, while PCS clearance showed greater benefit in those with longer vintage. However, these results require confirmation in larger independent cohorts.

### Nonlinear relationship between dialysis vintage and PCS clearance

**FIGURE 4:**
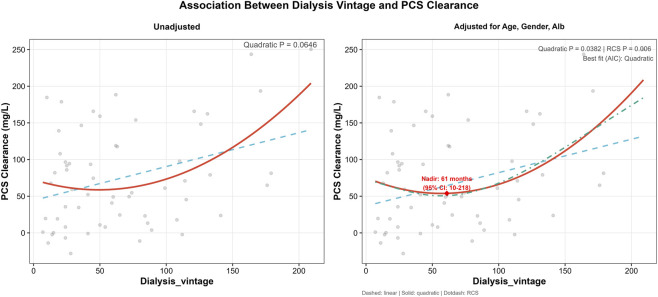
Association between dialysis vintage and PCS clearance. Scatter plots with regression lines illustrate the relationship between dialysis vintage (months) and PCS clearance (mg/L). Left panel: Unadjusted analysis. Dashed blue line: linear fit; Solid red line: quadratic fit. Right panel: Analysis adjusted for age, gender, and ALB. Dashed blue line: Linear fit. Solid red line: Quadratic fit. Dotdash green line: RCS with three knots at the fifth, 50th, and 95th percentiles (11.0, 52.0, 171.2 months). The red diamond marker in the adjusted panel indicates the nadir of the quadratic curve (60.9 months, 95%CI from bootstrap: 9.6–218.1 months). *P*-values for nonlinearity: quadratic P = 0.038, RCS P = 0.006. Model comparison by AIC favored the quadratic model (Quadratic AIC = 674.5, RCS AIC = 675.4, Linear AIC = 677.4).

To characterize the nonlinear relationship between dialysis vintage and PCS clearance, we compared linear, quadratic, and RCS models with three knots (at the fifth, 50th, and 95th percentiles of dialysis vintage distribution: 11.0, 52.0, and 171.2 months), adjusting for age, gender, and ALB. The quadratic model revealed a significant U-shaped relationship (*P* = 0.038, Adjusted R^2^ = 0.233), with a theoretical nadir at 56.9 months. Bootstrap resampling (1,000 iterations) yielded a median nadir of 60.9 months (95% CI: 9.6–218.1 months) (Figure 4). The RCS analysis confirmed significant nonlinearity (*P* = 0.006). Model comparison using AIC favored the quadratic model (AIC = 674.5) over the RCS (AIC = 675.4) and linear (AIC = 677.4) models, indicating that a quadratic function adequately captures the nonlinear pattern without overfitting. Collectively, both analytical approaches demonstrate a significant U-shaped relationship between dialysis vintage and PCS clearance, with clearance declining during approximately the first 5 years of dialysis, reaching a nadir around 60 months, and then gradually increasing thereafter (Figure 4; Supplementary Table S4).

### Machine learning feature selection

LASSO and Elastic Net regression with leave-one-out cross-validation were used for feature selection. Given the modest sample size, these results are exploratory. For PCS clearance, both methods consistently selected age, dialysis vintage, treatment method, and CRP. Bootstrap stability analysis (200 resamples, threshold >80%) revealed that treatment method (88.0%), dialysis vintage (83.5%), and age (80.0%) exceeded the threshold, while CRP showed a selection frequency of 79.5%, just below the cutoff. For β2-MG and AGEs clearance, neither LASSO nor Elastic Net selected any predictor (R^2^ = 0.000), and no predictor exceeded the 80% stability threshold, suggesting limited predictability from the measured variables. For IS clearance, Hb was selected by both methods and demonstrated high stability (85.5%) (Supplementary Figures S2-S4).

### Sensitivity analyses

Sensitivity analyses adjusting for time-weighted blood flow and TNF-α yielded consistent results (partial η^2^ range: 0.166-0.170, Supplementary Table S5). Exclusion of extreme values (≥3 standard deviations) did not alter the results for PCS clearance (*P* = 0.008). ANCOVA adjustment for age, gender, ALB, and blood flow confirmed the findings (*P* = 0.0081). Bootstrap resampling produced a median *P*-value of 0.005 (95% CI: 0.000-0.390, Supplementary Table S6). Post-hoc power analysis indicated 75% power to detect the observed effect size (Cohen’s d = 0.78), with 23 patients per group to achieve 80% power (Supplementary Table S7). To exclude mathematical coupling or measurement error, random noise (5% of measured value) was added to pre-dialysis PCS concentrations (500 iterations). The U-shaped pattern remained highly stable. Thus, the U-shaped relationship is unlikely to be attributable to mathematical coupling or measurement error (Supplementary Table S8).

## Discussion

In this prospective cohort study of 60 MHD patients, HD + KHA130 significantly enhanced PCS clearance compared with HDF, an effect that remained robust after multivariable adjustment. Significant interactions between treatment method and dialysis vintage were observed for PCS, β2-MG, and IS clearance, indicating that dialysis vintage modifies treatment efficacy. The relationship between dialysis vintage and PCS clearance followed a U-shaped pattern, with a nadir at approximately 60 months. Additionally, machine learning confirmed that dialysis vintage and treatment methods as stable predictors of PCS clearance. Collectively, these findings suggest that adsorption-based therapies exert solute-specific effects modulated by dialysis vintage.

The differential clearance patterns across solutes reflect differences in their physicochemical properties and the mechanisms of removal. Conventional hemodialysis effectively clears small water-soluble solutes but is limited in its capacity to remove middle molecules and protein-bound toxins (Sánchez-Ospina et al., 2024). In the present study, significant treatment effects were observed only for PCS clearance, aligning with the recognized ability of adsorption to enhance removal of protein-bound solutes (Meyer et al., 2023). Recent reviews have emphasized that adsorption techniques have opened a new frontier in dialysis purification, effectively removing uremic toxins previously considered inaccessible due to protein binding (Morisi et al., 2025). Our results support this concept, demonstrating that the incremental benefit of adsorption is most pronounced for protein-bound solutes. In contrast, β2-MG showed no overall treatment effect in the full cohort, despite *in vitro* evidence that adsorption can enhance its removal (Portales-Castillo et al., 2020). However, significant interactions were observed for β2-MG and IS, with subgroup analyses suggesting greater benefits in patients with shorter dialysis vintage. Given the exploratory nature of the subgroup analyses, these findings warrant confirmation in future studies. This observation aligns with prior work demonstrating that middle molecule clearance is influenced by multiple factors, including residual renal function, convective volume, and treatment duration (Obi et al., 2018).

A central finding is that dialysis vintage significantly modifies the effect of adsorption-based therapies. For β2-MG and IS clearance, the benefit of adsorption diminished with increasing dialysis vintage. For PCS clearance, subgroup analyses exploratorily suggested a benefit of HD + KHA130 in patients with longer dialysis vintage; however, the formal interaction test did not reach statistical significance. These divergent patterns suggest that long-term dialysis alters protein binding, residual renal function, and systemic inflammation, differentially influence the removal of various solute classes. The finding that dialysis vintage modifies treatment effects aligns with evidence that the duration of dialysis exerts complex effects on patient outcomes (Tentori et al., 2013; Yan et al., 2025). A recent cross-sectional study of hemodialysis patients reported that longer dialysis vintage was associated with increased symptom burden but also with improvements in quality of life across several domains (Yan et al., 2025). This paradox (worse physiological parameters but better patient-reported outcomes) parallels our observation that clearance patterns shift with vintage, suggesting that long-term dialysis may induce adaptive changes that influence both clinical outcomes and treatment responsiveness. Similarly, a large multicenter study in 1,131 hemodialysis patients demonstrated that abnormal Ca-P metabolism combined with longer dialysis vintage accelerated biological aging (Zhang et al., 2023), supporting the concept that dialysis vintage is not merely a temporal marker but an active modulator of pathophysiology.

The U-shaped relationship between dialysis vintage and PCS clearance was robustly demonstrated by both quadratic and RCS analyses, with consistent nadir estimates (quadratic: 56.9 months; bootstrap median: 60.9 months). The initial decline in clearance during the first approximately 5 years of dialysis may reflect progressive loss of residual kidney function, a major determinant of PBUT plasma concentrations, as PBUTs are predominantly excreted by renal tubular secretion rather than glomerular filtration (Lowenstein and Grantham, 2017; van Gelder et al., 2020). The loss of RKF over time is associated with worsening biochemical parameters and increased PBUT accumulation (Lowenstein and Grantham, 2017). The subsequent increase in clearance after approximately 60 months requires cautious interpretation. Several alternative explanations warrant consideration. First, adaptive changes in dialysis delivery over time, such as increased blood flow or treatment time, could enhance clearance. Second, survivorship bias may contribute: patients who survive to long dialysis vintage may represent a healthier subset with better-preserved clearance capacity, whereas those with poor clearance may have died earlier (Pickering, 2011). Third, regression to the mean could also play a role, as patients with unusually low clearance at the nadir may show higher values on subsequent measurements simply due to random variation rather than a genuine biological process (Affognon, 2025). Given the cross-sectional nature of our clearance measurements, we cannot distinguish between these possibilities. Given the cross-sectional nature of our measurements, we cannot distinguish between these possibilities. Nevertheless, the pattern explains why HD + KHA130 showed greatest benefit in long-vintage patients: when baseline clearance is lowest (around 60 months), the incremental benefit of adsorption is greatest.

The LASSO and Elastic Net analyses provided exploratory feature selection results. Given the modest sample size, we performed bootstrap stability analysis (200 resamples) with a predefined threshold of >80% selection frequency to identify robust predictors. For PCS clearance, treatment method (88.0%), dialysis vintage (83.5%), and age (80.0%) exceeded this threshold, while CRP (79.5%) fell just below. For IS clearance, Hb reached 85.5% and was considered stable. For β2-MG and AGEs clearance, no predictor exceeded the 80% threshold, and the R^2^ values were 0.000, indicating that the measured variables had limited predictive value for these solutes. Consistent with prior studies employing penalized regression methods in hemodialysis populations, our machine learning approach provided an unbiased assessment of predictor importance. Notably, the predictors identified by machine learning for PCS largely overlapped with those from conventional multivariable analysis, reinforcing the consistency of our findings. However, these machine learning results are exploratory and require validation in larger independent cohorts.

Several limitations should be acknowledged. The sample size limits the statistical power, particularly for subgroup analyses, which are exploratory and should be interpreted cautiously. The single-center design and cross-sectional clearance measurements limit generalizability and prelude assessment of longitudinal changes. Although we adjusted for multiple covariates; residual confounding remains possible. We did not measure residual renal function, which likely contributes to clearance, particularly in patients with shorter dialysis vintage. Low-molecular-weight heparin (60–80 U/kg) was used for anticoagulation, but anti-Xa activity was not monitored. Hemoperfusion cartridges may non-specifically bind heparin, potentially reducing effective anticoagulation, although no overt circuit clotting was observed; subclinical reductions cannot be excluded and we cannot rule out differential heparin adsorption between the HA130 and KHA130 or between the adsorption and HDF groups. Future studies should incorporate anti-Xa monitoring or fixed heparin protocols. Finally, while the observed improvements in PCS clearance (approximately 45 mg/L) were statistically significant, its clinical significance is unknown. Currently, no established minimal clinically important difference (MCID) exists for PBUT clearance, and its link to hard clinical endpoints (e.g., cardiovascular events, mortality) has not been quantified. Therefore, our findings should be interpreted as evidence of improved short-term clearance (a surrogate endpoint) rather than direct predictors of clinical outcomes. Future adequately powered trials linking PBUT clearance to patient-relevant endpoints are needed to establish clinical benefit.

In this study of MHD patients, HD + KHA130 significantly improved PCS clearance compared with HDF, an effect that remained robust after multivariable adjustment. Dialysis vintage emerged as a critical effect modifier, with distinct interaction patterns for protein-bound and middle molecule solutes. The U-shaped relationship between dialysis vintage and PCS clearance, with a nadir at approximately 60 months, provides mechanistic insight into why the benefit of adsorption is most pronounced in patients with longer dialysis duration. These findings suggest that treatment selection may need to be personalized based on both the targeted solute and the patient’s dialysis vintage, and they support the potential role of adsorption-based therapies in optimizing protein-bound toxin removal. Future studies should examine whether these clearance improvements translate into clinical benefits and should explore the mechanisms underlying the nonlinear relationship between dialysis duration and solute removal efficiency.

## Data Availability

The original contributions presented in the study are included in the article/Supplementary Material, further inquiries can be directed to the corresponding author.
