# Supplementary material for: Dialysis vintage modifies the effect of adsorption-based therapies on protein-bound toxin clearance
Source: Front Cell Dev Biol. 2026 May 29;14:1856227. doi: 10.3389/fcell.2026.1856227 (PMC13260634; doi:10.3389/fcell.2026.1856227)
Supplement: Supplementary file 1 [file DataSheet1.docx]

**Table S1 Univariate analysis of predictors for solute clearance (Top 5)**

**PCS Clearance**

| Variable | Coefficient | SE | *P* | FDR |
| --- | --- | --- | --- | --- |
| Dialysis vintage | 0.455 | 0.169 | 0.007 | 0.192 |
| Age | -1.120 | 0.728 | 0.124 | 0.824 |
| AGEs pre | -2.502 | 1.793 | 0.163 | 0.824 |
| CRP pre | -1.198 | 0.861 | 0.164 | 0.824 |
| Secondary hyperparathyroidism | 24.472 | 19.801 | 0.216 | 0.824 |

**β2-MG Clearance**

| Variable | Coefficient | SE | *P* | FDR |
| --- | --- | --- | --- | --- |
| Gender | 4.855 | 2.074 | 0.019 | 0.519 |
| Blood flow | -0.087 | 0.042 | 0.039 | 0.531 |
| Calcium | 10.688 | 5.874 | 0.069 | 0.620 |
| Hypertension | -3.577 | 2.257 | 0.113 | 0.729 |
| Anticoagulant dose | -0.002 | 0.001 | 0.135 | 0.729 |

**AGEs Clearance**

| Variable | Coefficient | SE | *P* | FDR |
| --- | --- | --- | --- | --- |
| Gender | 3.268 | 1.855 | 0.078 | 0.855 |
| Renal osteodystrophy | 5.358 | 3.274 | 0.102 | 0.855 |
| β2-MG pre | 0.166 | 0.105 | 0.114 | 0.855 |
| Age | 0.107 | 0.075 | 0.153 | 0.855 |
| Cardiovascular disease | 3.531 | 2.550 | 0.166 | 0.855 |

**IS Clearance**

| Variable | Coefficient | SE | *P* | FDR |
| --- | --- | --- | --- | --- |
| Hemoglobin | -0.264 | 0.113 | 0.020 | 0.528 |
| Phosphorus | -6.403 | 3.896 | 0.100 | 0.773 |
| Anemia | 6.601 | 4.045 | 0.103 | 0.773 |
| TNF-α pre | -0.147 | 0.095 | 0.123 | 0.773 |
| Hyperphosphatemia | -5.335 | 3.817 | 0.162 | 0.773 |

**Table S2 Overall treatment effect**

| Outcome | F-value | *P* |
| --- | --- | --- |
| PCS clearance | 5.843 | 0.005 |
| β2‑MG clearance | 0.487 | 0.621 |
| AGEs clearance | 0.006 | 0.994 |
| IS clearance | 1.789 | 0.176 |

**Table S3 Variance inflation factor assessment**

| Toxin | Max VIF | Mean VIF | VIF > 5 |
| --- | --- | --- | --- |
| PCS | 1.89 | 1.48 | 0 |
| β2-MG | 1.94 | 1.51 | 0 |
| AGEs | 1.89 | 1.49 | 0 |
| IS | 1.95 | 1.52 | 0 |

Note: VIF < 5 indicates no substantial multicollinearity.

**Table S4. Model comparison for the association between dialysis vintage and PCS clearance**

| Model | AIC | BIC | ΔAIC | P for nonlinearity | R² |
| --- | --- | --- | --- | --- | --- |
| Linear | 677.4 | 689.9 | 2.8 | Reference | 0.169 |
| Quadratic | 674.5 | 689.2 | 0 | 0.038 | 0.233 |
| RCS (3 knots) | 675.4 | 690.1 | 0.9 | 0.006 | — |

Note: Knots placed at the 5th, 50th, and 95th percentiles of dialysis vintage distribution (11.0, 52.0, and 171.2 months). Lower AIC indicates better model fit; ΔAIC = AIC - minimum AIC. RCS, restricted cubic spline.

**Table S5. Sensitivity analyses of adjusted effect sizes (partial η²) for PCS clearance**

| Covariates | Partial η² | 95% CI | P value | Interpretation |
| --- | --- | --- | --- | --- |
| Age + Gender + Alb | 0.165 | 0.032-0.407 | 0.0077 | Large |
| + Time-weighted blood flow | 0.167 | - | 0.0081 | Large |
| + TNF-α | 0.168 | - | 0.0076 | Large |
| + Blood flow + TNF-α | 0.170 | - | 0.0080 | Large |

Note: Bootstrap 95% confidence intervals were calculated only for the primary model (500 iterations). All models adjusted for age, gender, and albumin; dialysis vintage was not included as an effect modifier.

**Table S6. Sensitivity analysis for the primary treatment effect (PCS clearance)**

| Toxin | Analysis | Original *P* | Sensitivity *P* | Change |
| --- | --- | --- | --- | --- |
| PCS | Exclude outliers | 0.008 | 0.008 | Stable |
|  | Adjusted covariates | 0.008 | 0.008 | Stable |
|  | Bootstrap validation | 0.008 | 0.005 (0.000-0.390) | Stable |
| β2-MG | Exclude outliers | 0.651 | 0.514 | Stable |
|  | Adjusted covariates | 0.651 | 0.628 | Stable |
|  | Bootstrap validation | 0.651 | 0.389 (0.004-0.950) | Stable |
| AGEs | Exclude outliers | 0.994 | 0.994 | Stable |
|  | Adjusted covariates | 0.994 | 0.994 | Stable |
|  | Bootstrap validation | 0.994 | 0.515 (0.028-0.968) | Stable |
| IS | Exclude outliers | 0.182 | 0.182 | Stable |
|  | Adjusted covariates | 0.182 | 0.199 | Stable |
|  | Bootstrap validation | 0.182 | 0.105 (0.000-0.773) | Stable |

Note: Values are P-values from ANOVA (primary) or from sensitivity analyses. Bootstrap results presented as median (95% CI from 500 iterations). Adjusted for age, gender, ALB, and time-weighted blood flow.

**Table S7. Post-hoc Power Analysis**

| Parameter | Value |
| --- | --- |
| Detected effect size (Cohen's d) | 0.78 |
| Required sample size per group (80% power) | 23 |
| Total required sample size | 69 |
| Current sample size per group | 20 |
| Current statistical power | 75.4% |

Note: Calculated for PCS clearance comparing HDF vs HD+KHA130, α = 0.05, 3 groups.

**Table S8. Sensitivity analysis for mathematical coupling**

| Parameter | Original Analysis | Noise Injection (Median, 95% CI) |
| --- | --- | --- |
| Quadratic coefficient | 0.00688 | 0.00687 (0.00653-0.00722) |
| Nadir (months) | 58.3 | 58.3 (56.2-60.5) |
| P for nonlinearity | 0.036 | 99.8% of iterations P < 0.05 |
| R² | 0.227 | — |

Note: Random noise (5% of measured value) was added to pre‑dialysis PCS concentrations for 500 iterations. Absolute clearance was recalculated each iteration, and a quadratic model adjusted for age, gender, and albumin was fitted.


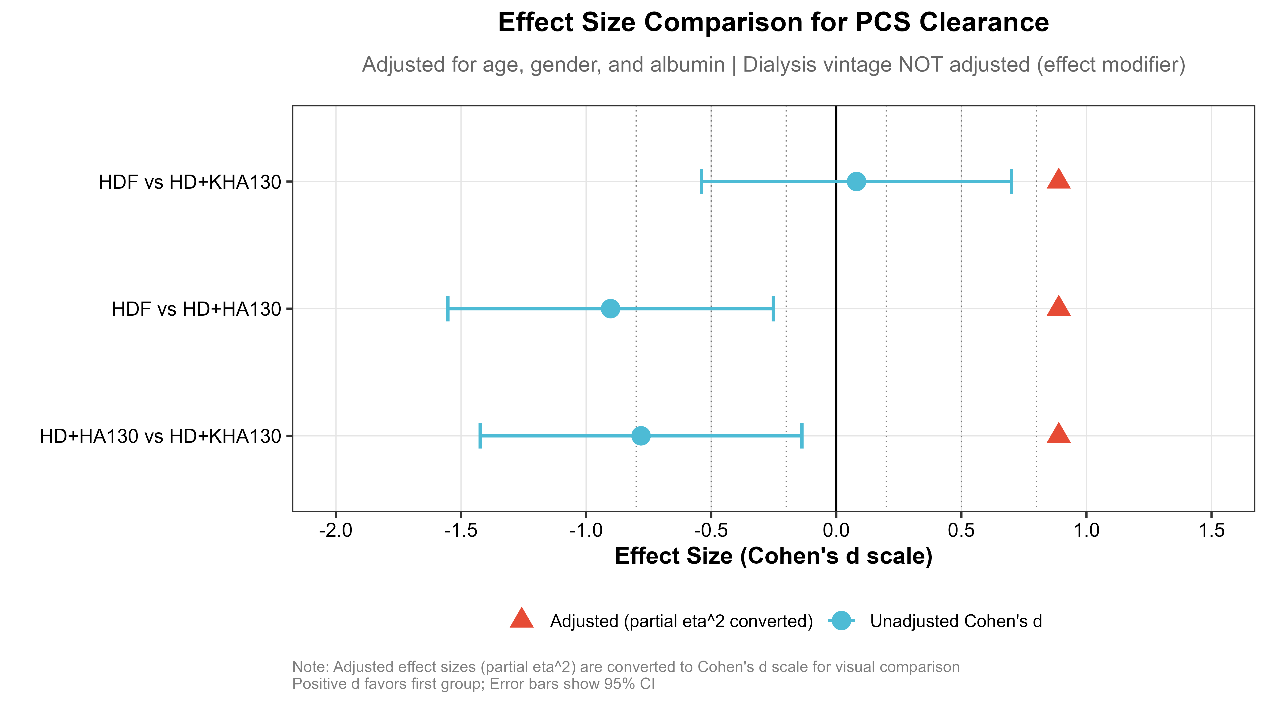


**Figure S1. Comparison of unadjusted and adjusted effect sizes for PCS clearance.** This figure compares the unadjusted Cohen's d (blue) with the adjusted effect size (red, partial η² converted to Cohen's d scale) for pairwise comparisons of PCS clearance across treatment groups. Unadjusted Cohen's d: Calculated directly from raw clearance values. Error bars represent 95% confidence intervals. Adjusted effect size: Derived from ANCOVA models adjusted for age, gender, and ALB. Partial η² (0.165, 95% CI: 0.032-0.407) was converted to Cohen's d scale using the formula: d = √(η²/(1-η²)) × 2. As ANCOVA provides a single effect size for the overall treatment effect (all three groups), the same converted value is shown for all pairwise comparisons for visual reference. Effect size interpretation (Cohen's d thresholds): d ≥ 0.2: small effect; d ≥ 0.5: medium effect; d ≥ 0.8: large effect.


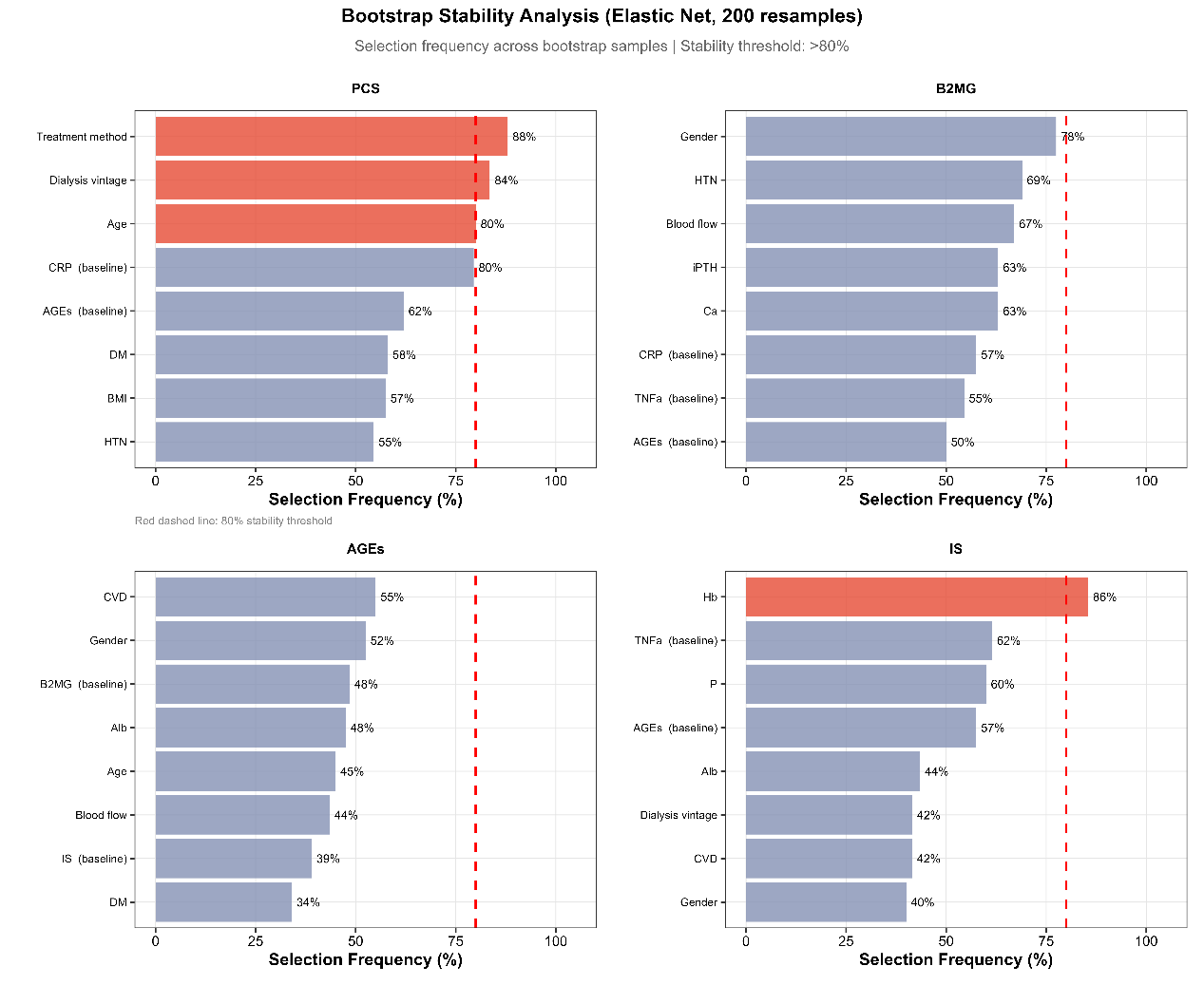


**Figure S2 Bootstrap stability of feature selection for solute clearance.** Bar plots display the selection frequency (%) of clinical and biochemical predictors for four uremic solutes (PCS, β2‑MG, AGEs, IS), derived from Elastic Net regression with 200 bootstrap resamples. The proportion of bootstrap samples in which each predictor was retained by the Elastic Net model, reflecting the robustness of its association with solute clearance. Predictors are ranked in descending order of selection frequency within each solute panel. Higher frequencies indicate greater stability and consistent predictive importance across resamples.


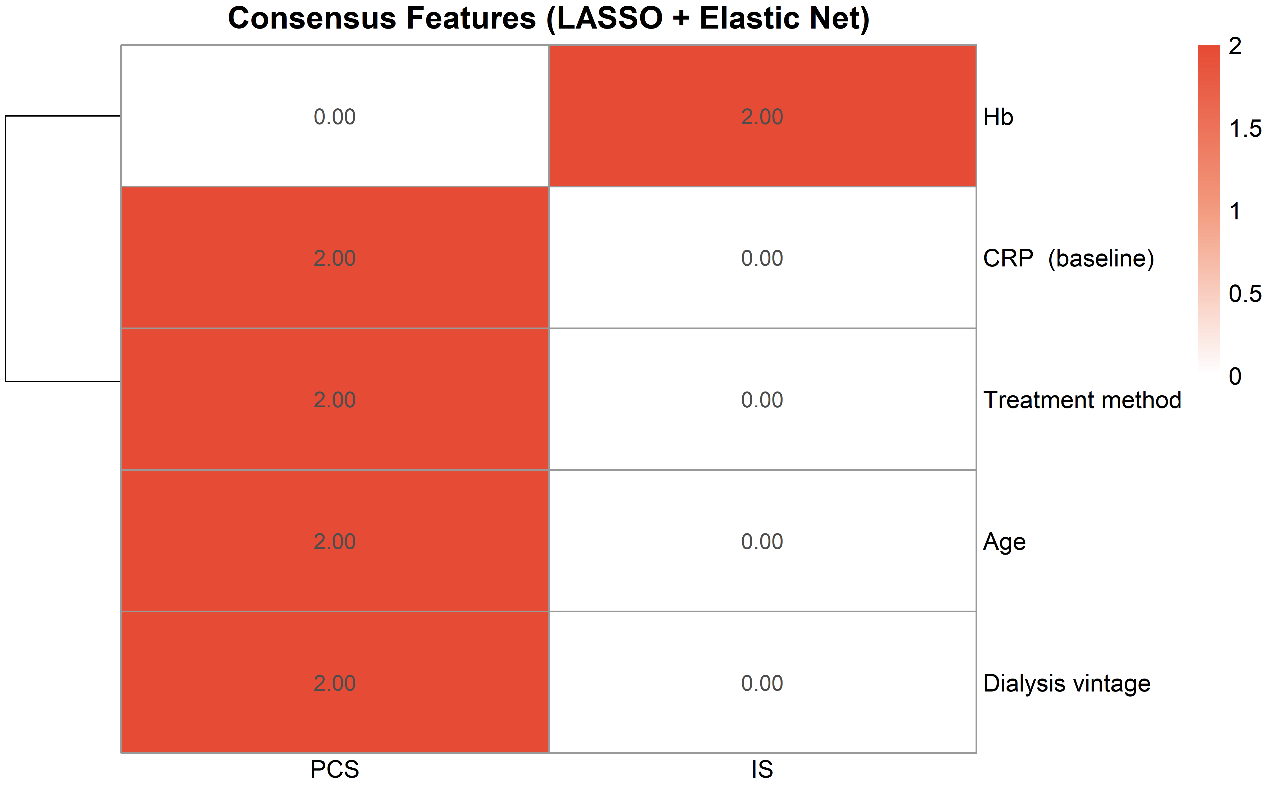


**Figure S3 Consensus feature selection for solute clearance.** Heatmap displays the consensus predictors of clearance for three uremic solutes (PCS, β2‑MG, IS) identified by both LASSO and Elastic Net regression models. A value of 2.00 indicates a predictor was selected by both LASSO and Elastic Net (consensus selection), while 0.00 indicates it was not selected by either model. Red shading (value = 2.00) highlights consensus predictors; white cells (value = 0.00) represent non-selected predictors. Consensus selection reflects robust predictive importance, as predictors were retained across two distinct regularized regression methods.


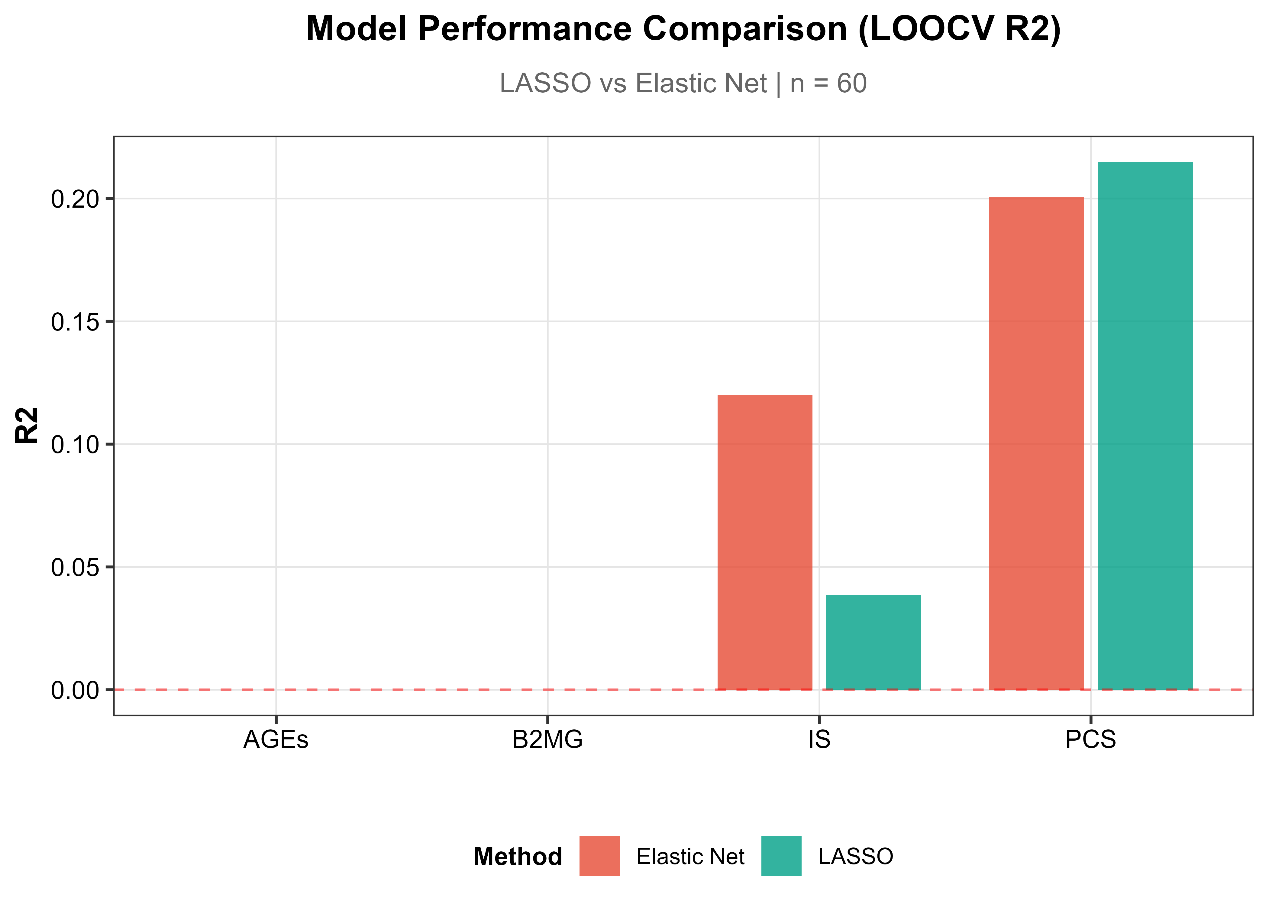


**Figure S4 Comparison of LASSO and Elastic Net model performance for solute clearance prediction.** Bar plots display the leave-one-out cross-validation (LOOCV) coefficient of determination (R²) for two regularized regression methods (LASSO: teal; Elastic Net: red) across four uremic solutes: advanced glycation end products (AGEs), β₂-microglobulin (β2‑MG), indoxyl sulfate (IS), and p-cresyl sulfate (PCS). Quantify the proportion of variance in solute clearance explained by each model, with higher values indicating better predictive performance. The horizontal dashed red line at R² = 0 represents a model with no explanatory power.
